# Using the Tg(*nrd:egfp*)*/albino* Zebrafish Line to Characterize *In Vivo* Expression of *neurod*

**DOI:** 10.1371/journal.pone.0029128

**Published:** 2012-01-03

**Authors:** Jennifer L. Thomas, Margaret J. Ochocinska, Peter F. Hitchcock, Ryan Thummel

**Affiliations:** 1 Department of Anatomy and Cell Biology and Department of Ophthalmology, Wayne State University School of Medicine, Detroit, Michigan, United States of America; 2 Department of Ophthalmology and Visual Sciences, University of Michigan Kellogg Eye Center, Ann Arbor, Michigan, United States of America; Center for Regenerative Therapies Dresden, Germany

## Abstract

In this study, we used a newly-created transgenic zebrafish, Tg(*nrd:egfp*)*/albino*, to further characterize the expression of *neurod* in the developing and adult retina and to determine *neurod* expression during adult photoreceptor regeneration. We also provide observations regarding the expression of *neurod* in a variety of other tissues. In this line, EGFP is found in cells of the developing and adult retina, pineal gland, cerebellum, olfactory bulbs, midbrain, hindbrain, neural tube, lateral line, inner ear, pancreas, gut, and fin. Using immunohistochemistry and *in situ* hybridization, we compare the expression of the *nrd:egfp* transgene to that of endogenous *neurod* and to known retinal cell types. Consistent with previous data based on *in situ* hybridizations, we show that during retinal development, the *nrd:egfp* transgene is not expressed in proliferating retinal neuroepithelium, and is expressed in a subset of retinal neurons. In contrast to previous studies, *nrd*:egfp is gradually re-expressed in all rod photoreceptors. During photoreceptor regeneration in adult zebrafish, *in situ* hybridization reveals that *neurod* is not expressed in Müller glial-derived neuronal progenitors, but is expressed in photoreceptor progenitors as they migrate to the outer nuclear layer and differentiate into new rod photoreceptors. During photoreceptor regeneration, expression of the *nrd:egfp* matches that of *neurod*. We conclude that Tg(*nrd:egfp*)/*albino* is a good representation of endogenous *neurod* expression, is a useful tool to visualize *neurod* expression in a variety of tissues and will aid investigating the fundamental processes that govern photoreceptor regeneration in adults.

## Introduction

NeuroD is a basic helix-loop-helix (bHLH) transcription factor that plays a common role in persistently mitotic cells as an essential link between cell cycle exit, cell fate determination, and cell survival [1]. In the vertebrates, *neurod* is expressed in areas of the brain including the cortex, cerebellum, olfactory bulb, eye, and midbrain [1], [2], [3], [4]. *Neurod* is also expressed in the developing endocrine pancreas [5], the auditory and vestibular neuroblasts of the developing inner ear [6], and the lateral line of teleost fish [7]. In both mice and zebrafish, *neurogenin* is expressed in cells prior to *neurod*, [2], [4] and overexpression of the *neurogenin* homolog in Xenopus (*X-NGNR-1*) induces ectopic expression of *Xneurod* mRNA [8], suggesting that *neurogenin* is an upstream regulator of *neurod* . During both zebrafish and mammalian retinogenesis, *neurod* is first expressed in retinal neuroepithelial cells as they exit the cell cycle. Once distinct cell types have formed, *neurod* is expressed in a subset of cells in both the inner nuclear layer (INL) and outer nuclear layer (ONL), but not in the ganglion cell layer (GCL) [1], [9]. By adulthood, *neurod* expression was previously reported to persist in a subset of amacrine cells nascent cone photoreceptors near the retinal margins [1], [10].

NeuroD functions in both neuronal and non-neuronal tissues and its specific role appears to be dependent of the mitotic state of the cell. In mitotic cells, NeuroD specifically regulates proliferation [11], [12] and cell cycle exit [13]. This was first demonstrated in Xenopus embryos where ectopic expression of *Xneurod* results in premature differentiation of neuronal precursors [11]. In post-mitotic cells, loss of NeuroD function can result in cell death during after cell differentiation [12], [14], [15], [16]. For example, NeuroD-null mice are deaf due to apoptosis of the otic epithelium and neurons that form the cochlear-vestibular ganglion [14]. In addition, loss of NeuroD in mice also causes age-related rod photoreceptor degeneration [16].

During mouse retinogenesis, *neurod* expression in retinal progenitors promotes the genesis of neurons versus glial cells, and specifically promotes amacrine cell fates versus bipolar cell fates [9], [17]. In the developing chick retina, NeuroD is necessary and sufficient for photoreceptor differentiation [18], [19]. During zebrafish retinogenesis, NeuroD regulates exit from the cell cycle among late-stage photoreceptor progenitors [20].

The zebrafish is a unique model because of its ability fully regenerate a variety of tissues, including the fin [21], [22], heart [23], spinal cord [24] and retina [25]. Numerous approaches have been developed to induce retinal regeneration, including cytotoxins [26], [27], [28], laser ablation [29], stab wound [30] and constant intense light treatment, which selectively kills rod and cone photoreceptors [25], [31]. Whereas each of these methods is unique in its severity of injury and selectivity of cellular damage, the mechanisms of regeneration are conserved. Cell death elicits a subset of Müller glial cells to reenter the cell cycle and generate retinal progenitors that differentiate into all the retinal cell types lost to the original injury [25], [32].

In this study, the Tg(*nrd:egfp*)*/albino* zebrafish line was used to characterize *neurod* expression. In this line, the transgene is expressed in the CNS, including the retina, olfactory bulbs, midbrain, hindbrain, neural tube, lateral line, inner ear and visceral organs, including the pancreas and gut. A detailed analysis of *neurod* expression, as evidenced by EGFP localization, is shown during retinal development in larvae and photoreceptor regeneration in adults. During regeneration we show that the *neurod* transgene in not expressed in Müller glial cells as they reenter the cell cycle, nor is it expressed in their immediate progeny. However, the transgene is expressed in progenitors of the regenerating photoreceptors as they exit the cell cycle and begin differentiating. We find that this *neurod* transgene is a useful tool to visualize *neurod* expression during the development of multiple organ systems and during the dynamic process of adult retinal regeneration.

## Materials and Methods

### Ethics Statement

All protocols used in this study were approved by the animal use committee at the University of Notre Dame and Wayne State University School of Medicine (Protocol # A040310) and are in compliance with the ARVO statement for the use of animals in vision research.

### The Tg(*nrd:egfp*) line and zebrafish maintenance

The Tg(*nrd:egfp*) line was obtained as a gift from Alex Nechiporuk, who generated the line [33]. Briefly, a BAC clone (dK33b12) was isolated that contained 67 kilobase pairs (kb) of sequence upstream and 89 kb of sequence downstream of *neurod*. Recombineering resulted in *egfp* positioned at the endogenous start site. This construct (ZFIN ID: ZDB-TGCONSTRCT-080701-1) was used to make transgenic animals. Adult fish positive for the transgene were out-crossed to *albino* mutants. Fish were fed a combination of brine shrimp and flake food three times daily and maintained under a daily light cycle of 14 hours light (250 lux):10 hours dark at 28.5°C [34].

### Constant intense-light treatment protocol

Photoreceptor degeneration was accomplished by constant intense-light treatment as previously described [25]. Adult Tg(*nrd:egfp*)/*albino* zebrafish were subjected to dark adaptation for 10 days, and then transferred to a clear 1.8 liter tank positioned between 4 halogen lamps (250 watts). The fish were continuously exposed to the light (8000 lux) for up to four days, at which time they were returned to standard light/dark conditions. During the light treatment, water temperature remained between 30–33° C.

### EdU labeling of retinal progenitors

5′-ethynyl-2′-deoxyuridine (EdU; Invitrogen, Carlsbad, CA) was diluted in 1XPBS to 1 mg/mL and injected intraperitoneally (50 microliters) into adult Tg(*nrd:egfp*)/*albino* zebrafish. Two injection protocols were used. In order to label all of the progenitors, daily injections were performed throughout the light treatment [35]. In order to label a subset of the progenitors, a single injection was performed immediately prior to starting the light treatment. Eyes were harvested 96 hours after light onset and processed for immunohistochemistry as described below. For EdU immunolocalization, Click-iT EdU AlexaFluor 594 Imaging Kit was performed per the manufacturer's instructions (Invitrogen), followed by EGFP immunolocalization as described below.

### Wholemount brightfield and fluorescent imaging

Live transgenic embryos and adult fish were anesthetized with 2-phenoxyethanol prior to microscopy. Images were captured on a Spot digital camera (Diagnostic Instruments; Sterling Heights, MI, USA) attached to a Leica M165 FC stereomicroscope.

### Immunohistochemistry and microscopy

Tg(*nrd:egfp*)/*albino* zebrafish were collected at 24, 32, 42, 48, 72, and 96 hour post-fertilization (hpf), dechorionated (if necessary), and fixed in either 4% paraformaldehyde in 5% sucrose/1× PBS or 9∶1 ethanolic formaldehyde (100% ethanol: 36% formaldehyde) overnight at 4° C. Embryos and larva were cryoprotected in 5% sucrose/1× PBS twice at room temperature, followed by a 30% sucrose/1× PBS wash overnight at 4° C. Larvae were frozen in Tissue Freezing Medium (TFM) (Triangle Biomedical Sciences, Durham, NC) and cryosectioned at 18 µm. Sections were transferred to glass slides, dried for up to 4 hours at 56° C, and stored at −80°C.

For controls and those receiving photolytic lesions, fish were euthanized and their eyes were harvested at various times after light onset: 0, 42, 72, or 96 hours, or 7 or 11 days. Eye tissue was fixed in either 4% paraformaldehyde in 5% sucrose/1× PBS or 9∶1 ethanolic formaldehyde (100% ethanol: 36% formaldehyde) overnight at 4° C, cryoprotected and embedded in TFM . Eyes were cryosectioned at 18 µm and sections were transferred to glass slides, dried at 56° C for 2 hours, and stored at −80° C.

Immunohistochemistry was performed as previously described [32]. The following primary antibodies and dilutions were used: chicken anti-insulin polyclonal antisera (1∶200, Abcam, Cambridge, MA) mouse monoclonal anti-green fluorescent protein (GFP) antibody (1∶200, Sigma Chemical, St. Louis, MO), mouse monoclonal anti-PCNA antibody (1∶500, Sigma Chemical, St. Louis, MO), rabbit polyclonal anti-PCNA antisera (1∶100, AnaSpec, Fremont, CA), mouse monoclonal anti-glutamine synthetase antibody (1∶500, Chemicon International, Temecula, CA), mouse monoclonal anti-HuC/D (1∶30, Invitrogen), mouse monoclonal anti-Zpr-3 antibody (1∶200, Zebrafish International Resource Center, Eugene, OR), and mouse monoclonal anti-Zpr-1 antibody (1∶200). Secondary antibodies used for this study included goat anti-mouse 488 and 594, goat anti-rabbit 488 and 594, and goat anti-chicken 594 (Invitrogen, Carlsbad, CA). In addition, nuclei were labeled using TO-PRO-3 (1∶750, Invitrogen).

Confocal microscopy was performed using a Leica TCS SP2. Approximately 12–15 retinal sections taken at or adjacent to the optic nerve were examined for each time point.

### RNA *in situ* hybridization and subsequent immunohistochemistry

For *in situ* hybridizations, eyes were dissected and preserved (as described above), cryosectioned at 10 µm and processed as described previously (Ochocinska and Hitchcock, 2009). Briefly, sections were rehydrated in decreasing concentrations of ethanol, permeabilized with Proteinase K, and treated with acetic anhydride to reduce non-specific binding of the probe. The 2,158 basepair Digoxygenin-labeled probe was synthesized from a full-length cDNA of *neurod* (kindly provided by Zhiyuan Gong, National University of Singapore) [2]. The probe was applied to the sections and incubated overnight at 55° C. Sections were then washed at 55° C to remove unbound probe, and processed for immunocytochemistry with antibodies against DIG that were conjugated to alkaline phosphatase. NBT/BCIP (Roche) was used as a substrate for the alkaline phosphatase. The reaction was stopped (generally after 1 hr) with Tris-HCl buffer at pH 8.0. The reaction product was preserved by briefly fixing the sections with 4% paraformaldehyde prior to the GFP immunohistochemistry (see above).

## Results

### Tg(*nrd:egfp*) expression is observed in multiple tissues from embryonic development through adulthood

**Figure 1:**
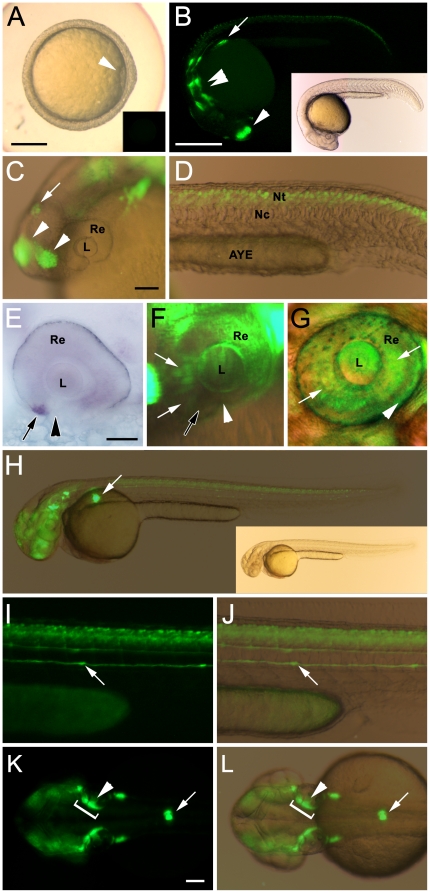
Wholemount brightfield and flourescent images showing *nrd:egfp* transgene expression in the developing Tg(*nrd:egfp*)*/albino* zebrafish. (A) Brightfield image with fluorescent inset showing the absence of transgene expression at 6 hpf. Arrowhead notes the gastrulation site (B) Fluorescent image with a brightfield inset at 24 hpf showing EGFP expression in the developing pancreas (arrow), olfactory bulbs (single arrowhead), and regions of the midbrain and hindbrain (double arrowheads). (C) High magnification overlay of brightfield and fluorescent images at 24 hpf. EGFP is detected in the olfactory bulbs (arrowheads), pineal gland (arrow), and inner ear (top right of panel). At this time it is not observed in the developing eye. (D) High magnification overlay of brightfield and fluorescent images at 24 hpf showing EGFP expression in the neural tube. (E) RNA *in situ* hybridization at 31 hpf, showing endogenous *neurod* expression in the ventral nasal patch (arrow), immediately adjacent to the choroid fissure (arrowhead). (F) Fluorescent image showing EGFP-positive cells in the retina at 32 hpf that are within a region (white arrows) immediately adjacent to the ventral nasal patch (black arrow). The choroid fissure is marked with a white arrowhead). (G) High magnification overlay of brightfield and fluorescent images at 48 hpf showing EGFP expression in throughout the inner retina (arrows) and in the outer retina (arrowhead). (H) Overlay of brightfield and fluorescent at 48 hpf (with brightfield image inset), showing EGFP in the developing pancreas. (I–J) Fluorescent (I) and brightfield overlay (J) of image shown in H. EGFP expression is observed in the neural tube and lateral line (arrow). (K) Fluorescent image of the dorsal head at 48 hpf. EGFP expression is observed in the pancreas (arrow), inner ear (arrowhead, with bracket to indicate location of ear), and regions of the CNS. (L) Corresponding overlay of brightfield and fluorescent images. Abbreviations: L (Lens), Re (Retina), AYE (Anal Yolk Extension), Nc (Notochord), Nt (Neural tube). Scale bar: 250 (A); 250 microns (B, H); 100 microns (C, D, I, J); 50 microns (E); 50 microns (K, L).

The expression of the *nrd:egfp* transgene was first examined by wholemount fluorescence microscopy. Consistent with previously submitted gene expression data of endogenous *neurod* [36], the transgene is not maternally expressed (data not shown), and was not observed during gastrulation at 6 hours post-fertilization (Fig. 1A). EGFP expression was first observed at 24 hours post-fertilization (hpf) in the olfactory bulbs, pineal gland, inner ear, midbrain, hindbrain, pancreas and neural tube (Fig. 1B, C), but was not observed in the developing eye (Fig. 1C). This expression pattern was identical to the previously reported expression pattern of endogenous *neurod* [36]. In the developing zebrafish retina, endogenous *neurod* was first observed in the ventral nasal patch at 31 hpf [1] (Fig. 1E), which coincides with the initiation of a ventral-to-dorsal wave of neurogenesis. At 32 hpf, very weak EGFP expression (note the over-saturation of the surrounding tissues) was observed in the retina immediately dorsal to the ventral nasal patch (Fig. 1F). At 48 hpf, EGFP-positive cells were observed throughout the inner retina (Fig. 1G, arrows) and outer retina (Fig. 1G, arrowhead), indicating that the wave of neurogenesis had completed. Persistent EGFP expression was also observed in areas of the central nervous system, lateral line, and the pancreas (Fig. 1H–L).

**Figure 2:**
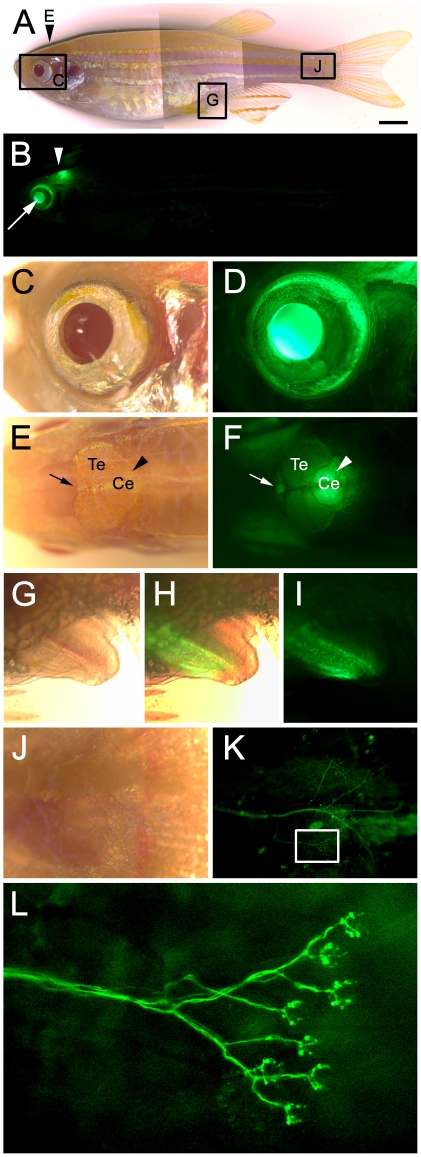
Wholemount brightfield and fluorescent images showing *nrd:egfp* trangene expression in adult Tg(*nrd:egfp*)*/albino* zebrafish. (A) A multiple brightfield image overlay showing the entire adult fish. Arrowhead indicates the location of the pineal gland and cerebelum (shown in Panels E and F). The boxes and corresponding panel letter indicate the location of the higher magnification images shown in Panels C–K. (B) Corresponding fluorescent image to Panel A, showing EGFP expression in the eye (white arrow) and the pineal (white arrowhead). (C–D) Brightfield and corresponding fluorescent image showing EGFP expression in the eye. (E) Brightfield image of the dorsal side of the head showing the pineal gland (arrow), telecephalon (Te), and cerebellum (Ce, arrowhead). (F) Corresponding fluorescent image showing EGFP expression in the pineal gland (arrow) and cerebellum (arrowhead). (G–I) Brightfield, overlay, and fluorescent images of the anus and its expression of the transgene. (J–K) Brightfield and corresponding fluorescent image showing EGFP expression in nerves located near the girdle of the tail fin. The box indicates the location of image shown in Panel L. (L) A high magnification fluorescent image of a branch of the nerve shown in Panel K, most likely of the posterior lateral line, showing EGFP expression in each of the terminating synaptic buttons. Scale bar: 2 mm (A).

**Figure 3:**
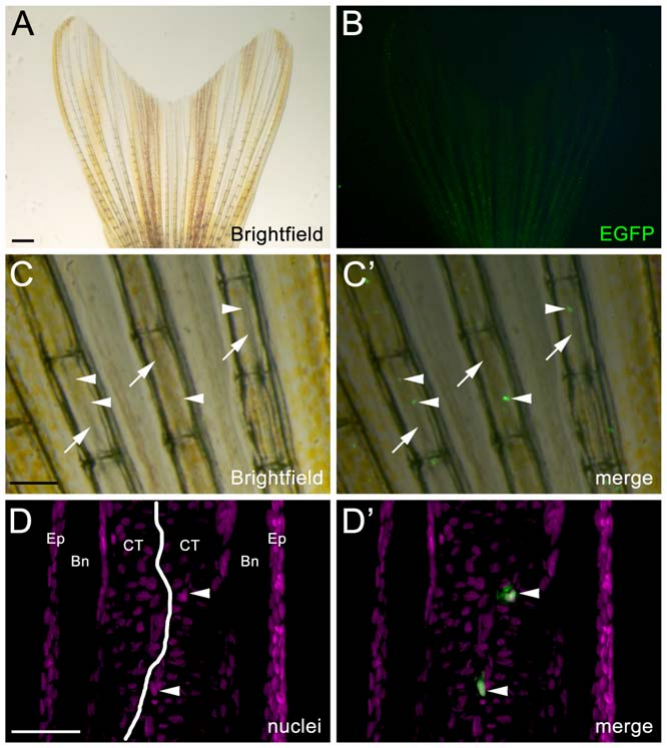
Wholemount brightfield and flourescent images showing *nrd:egfp* transgene expression in the adult caudal tail fin. (A) Brightfield image of the adult caudal fin. (B) Corresponding fluorescent image to panel A. EGFP expression is visualized in the nerve coursing through each bony hemiray of the caudal fin, however at this level of magnification, it is difficult to visualize. (C–C′) High magnification brightfield and corresponding fluorescent overlay showing multiple bony lepidotrichia. The arrows point to the nerve running within each bony hemiray and arrowheads point to EGFP-positive ganglia associated with the nerve. (D and D′) A section of a single bony ray immunolabeled with EGFP to show the transgene and co-labeled with TO-PRO-3 to show all nuclei (magenta). The white line in Panel D shows the location of the nerve. Ep = Epithelium, Bn = bony ray, CT = connective tissue.

**Figure 4:**
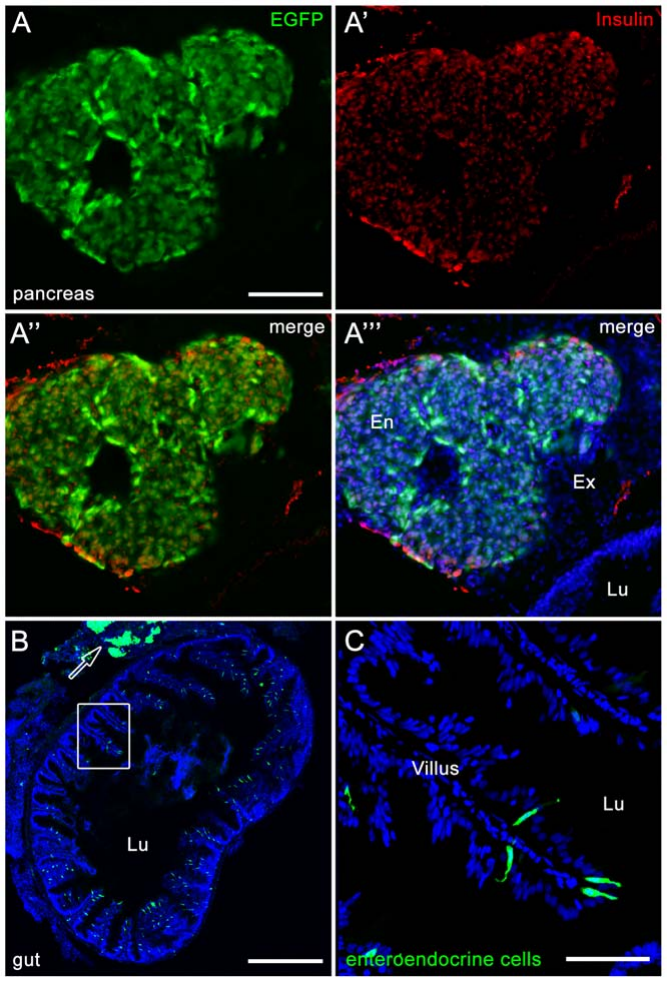
Section from Tg(*nrd:egfp*)*/albino* zebrafish showing *nrd:egfp* trangene expression in the endocrine pancreas (A–A′″) and gut (B–C). (A–A′″). Immunolocalization of EGFP (A, green) co-labels with Insulin (A′, red) in the endocrine pancreas (A′″, En). Co-labeling with TO-PRO-3 shows all nuclei (A′″, blue) and indicates the surrounding exocrine pancreas (A′″, Ex) and adjacent lumen of the gut (A′″, Lu). (B) EGFP expression can be visualized in enteroendocrine cells within each villus and in the surrounding smooth muscle. The adjacent pancreas is also visible (arrow). (C) High magnification image of the box in panel B.

In the adult zebrafish, we observed persistent and intense EGFP expression in the eye, pineal gland, and cerebellum (Fig. 2B, D and F). This is consistent with previous reports indicating expression of endogenous neurod in the adult pineal gland [2], [37] and cerebellum [38], [39]. Expression was also observed surrounding the anus (Fig. 2H and I). Closer examination of the zebrafish body revealed weak EGFP expression in an extension of the lateral line, which was especially visible near the tail fin girdle (Fig. 2J and K). This expression revealed intricate nerve arborization and synaptic boutons (Fig. 2K and L). In addition, EGFP expression was observed in ganglia associated with the nerve that extends through each bony hemiray of the caudal fin, which are anchored in the fin girdle and give support for fin structure (Fig. 3B, C′, D′). The transgene is not upregulated in the wound epithelium or proliferative blastema during fin regeneration, but is re-expressed in ganglia associated with the regenerating nerve (data not shown). In addition, EGFP was observed in the adult endocrine pancreas and in presumptive enteroendocrine cells in the gut epithelium. Specifically, EGFP co-labeled with Insulin in the endocrine pancreas, but was not expressed in the surrounding exocrine pancreas (Fig. 4A′″). Finally, EGFP was observed in a small number of cells within the intestinal epithelium (Fig. 4B and C). *Neurod* has previously been shown to be expressed in enteroendocrine cells and be required for proper enteroendocrine cell differentiation. Based on these data and the location, distribution, and morphology of the EGFP-positive cells observed in the gut, the transgene appears to label both endocrine cells of the pancreas and enteroendocrine cells in the adult gut.

### The *nrd:egfp* transgene is expressed in cells as they exit the cell cycle and in a subset of differentiated retinal neurons

**Figure 5:**
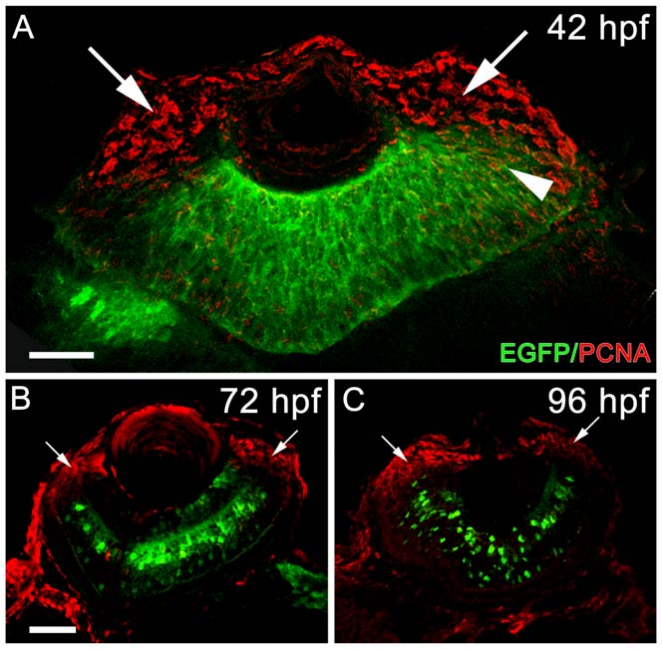
Retinal sections from embryonic Tg(*nrd:egfp*)*/albino* zebrafish immunolabeled with PCNA (red) and EGFP (green). (A) At 42 hpf, EGFP is detected throughout the retinal neuroepithelium in the central retina. PCNA immunolocalization, showing proliferating cells, is primarily restricted to the CMZ (arrows). In the overlapping region of PCNA and EGFP co-labeling can be visualized (arrowhead). (B) At 72 hpf, EGFP is detected in a subset of ganglion, amacrine and bipolar cells, and is not present in the outer nuclear layer. Proliferating cells are restricted to the CMZ (arrows), and there is no evidence of PCNA and EGFP co-immunolabeling. (C) At 96 hpf, there is persistent expression of EGFP detected in a subset of ganglion, amacrine and bipolar cells. Proliferating cells are restricted to the CMZ (arrows) and do not co-label with the transgene. Scale bars: 25 microns (A) and 50 microns (B, C).

During retinal development in zebrafish, *neurod* is required for photoreceptor progenitors to exit the cell cycle [20]. We examined expression of the *nrd:egfp* transgene in relationship to retinal progenitors immunolabled with Proliferating Cell Nuclear Antigen (PCNA), a marker for proliferating cells [25], [40]. At 42 hpf, we observed PCNA-positive cells restricted to the circumferential marginal zone (CMZ) and EGFP expression in the central retina with colocalization of cells in the overlapping regions of EGFP and PCNA expression (Fig.5A). Following retinal lamination, at 72 and 96 hpf, PCNA-positive cells were restricted to the CMZ and no longer colocalized with the transgene, and EGFP expression was seen in a subset of amacrine and bipolar cells (Fig. 5B and C).

**Figure 6:**
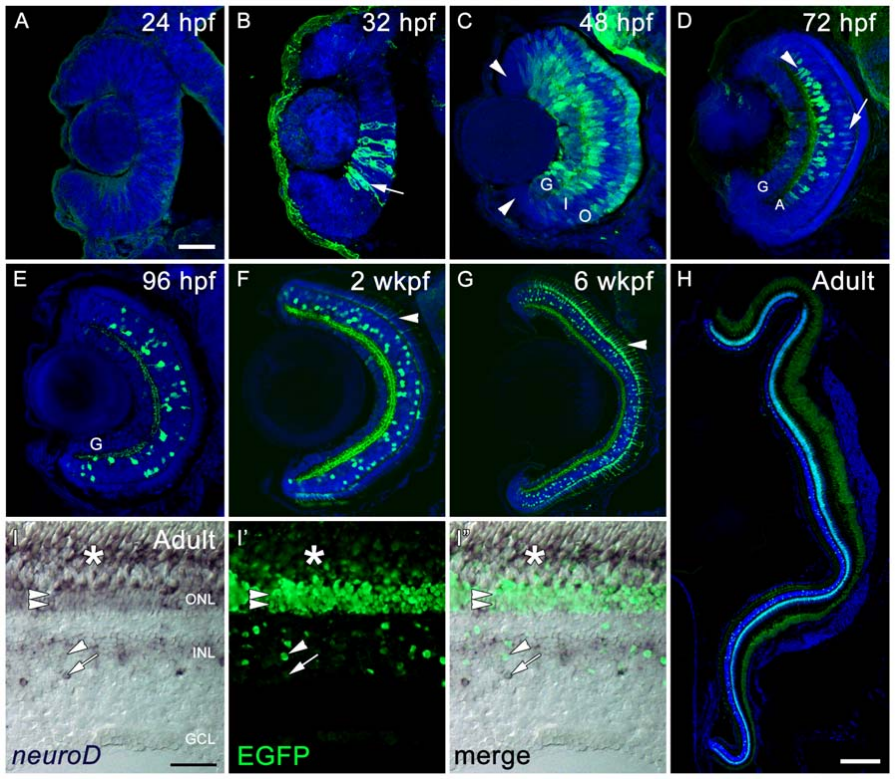
Retinal sections from embryonic Tg(*nrd:egfp*)*/albino* zebrafish showing EGFP in green and a nuclear stain, TO-PRO-3, in blue (A–H). All retinas are oriented with dorsal toward the top and ventral toward the bottom. RNA *in situ* hybridization in adult Tg(*nrd:egfp*)*/albino* retinas comparing endogenous expression of *neurod* to the transgene (I–I″). (A) At 24 hpf, EGFP is not detected in the retinal neuroepithelium. (B) At 32 hpf, EGFP is detected in a few cells immediately adjacent to the ventral nasal patch (arrow). (C) At 48 hpf, distinct retinal layers can be visualized. EGFP is detected in the ganglion cell layer (G), inner nuclear layer (I) at the level of the amacrine cells, and in the outer nuclear layer (O), but from this timepoint onward, is not detected in the CMZ (arrowheads). (D) At 72 hpf, EGFP expression is restricted to very few ganglion cells (G), a subset of amacrine (A) and bipolar cells (arrow), as well as the inner plexiform layer (arrowhead). (E) At 96 hpf, persistent EGFP expression is visible in a subset of amacrine and bipolar cells, in the inner plexiform layer, and very weakly expressed in a few ganglion cells (G). (F) At 2 wkpf, EGFP begins to reappear in a subset of rod photoreceptors (arrowhead). (G) At 6 wkpf, a majority of rod photoreceptors express EGFP as well as a subset of amacrine and bipolar cells, and the inner plexiform layer. (H) In the adult eye, all rod photoreceptors express EGFP, as well as a subset of amacrine and bipolar cells. (I–I′″) Comparing endogenous *neurod* (I) to transgenic *neurod* (I″) in the adult retina. Endogenous *neurod* is weakly expressed in rod photoreceptor soma in the outer nuclear layer (ONL, double arrowheads) and their corresponding rod inner segments (asterisk). Expression is also observed in individual neurons in the inner nuclear layer (INL). Strong EGFP was observed in the ONL (double arrowheads) and rod inner segments (above), and in individual neurons in the INL. Every EGFP-positive cell contains at least some endogenous *neurod*, but the levels vary greatly. Some cells have strong EGFP expression but weak endogenous *neurod* (single arrowhead), while other show the opposite expression profile (arrow). Scale bars: 50 microns (A–G), 300 microns (H), 50 microns (I–I′″).

Closer examination of the *nrd:egfp* transgene expression during retinal development and in adulthood revealed similarities and differences between EGFP expression and the previous report of endogenous *neurod* expression. Similar to the previous observation [1], EGFP expression was not observed in undifferentiated neuroepithelium 24 hpf (Fig. 6A) and at no age was EGFP observed in the retinal progenitors located in the circumferential marginal zone (CMZ) (Figs. 5 and 6). EGFP was first observed in the retina immediately adjacent to the ventral nasal patch at 32 hpf (Fig. 6B). EGFP expression expanded throughout the inner and outer retina at 48 hpf (Fig. 6C). At 72 hpf, endogenous *neurod* expression was reported to be expressed only in amacrine cells and in the ONL [1]. In contrast, EGFP was present in a subset of ganglion cells, amacrine cells, and bipolar cells, but was not detected in the ONL (Fig. 6D). In addition, the EGFP signal grew slowly in the population of rod photoreceptors, starting at 2 weeks post fertilization (wkpf) (Fig. 6F), and was present in all rod photoreceptors in adults (Fig. 6H). Although expression in the ONL and bipolar cells was not reported previously, we find that endogenous *neurod* is expressed in each of these cell types in adults (Fig. 6I–I″). Specifically, weak expression of *neurod* was observed in the ONL, with strong expression in the rod photoreceptor inner segments. In the INL, every EGFP-positive cell exhibited at least some *neurod* expression. However, many cells that were strongly expressing *neurod*, showed only weak EGFP, and vice versa, perhaps reflecting the dynamic regulation of *neurod* transcription in these neurons.

**Figure 7:**
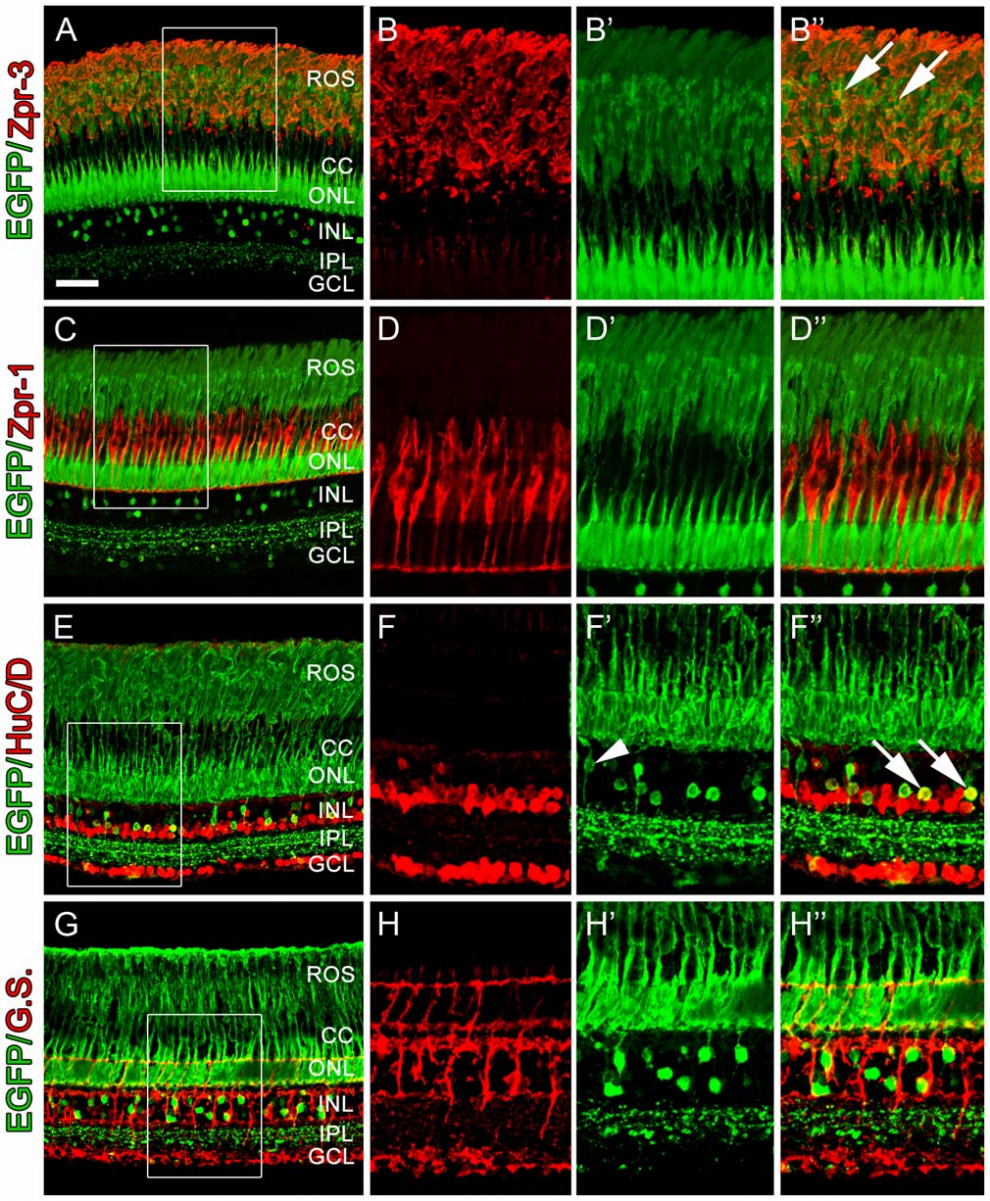
Retinal sections from adult Tg(*nrd:egfp*)/*albino* zebrafish. (A) Immunolocalization of GFP (green) to visualize the *nrd:egfp* transgene and Zpr-3 to visualize rod photoreceptors. (B) Higher magnification inset of (A) showing Zpr-3 immunolocalization in rods. (B′) Overlay image showing that the transgene is present in rod photoreceptors and co-labels with rod inner and out segments. (C) Immunolocalization of GFP (green) to visualize the *nrd:egfp* transgene and Zpr-1 (red) to visualize double cones. (D) Higher magnification inset of (A) showing Zpr-1 immunolocalization in double cones. (D′) Overlay image showing that EGFP is restricted to rod photoreceptor soma and outer segments and does not co-label with double cones. (E) Immunolocalization of GFP (green) to visualize the *nrd:egfp* transgene and HuC/D (red) to visualize all amacrine and ganglion cells. (F) Higher magnification inset of (C) showing HuC/D expression in amacrine and ganglion cells only. (F′) Overlay image showing co-labeling of EGFP with a subset of HuC/D-positive amacrine cells (arrows) and a HuC/D-negative bipolar cell extending its processes from the photoreceptors to the IPL (arrowhead). (G) Immunolocalization of GFP (green) to visualize the *nrd:egfp* transgene and Glutamine Synthetase (G.S.; red) to visualize all Müller glial cells. (H) Higher magnification inset of (E) showing G.S.-positive Müller glial cells. (H′) Overlay image showing that EGFP does not co-label with G.S.-positive Müller glial cells. ROS, rod outer segments; CC, cone cells; ONL, outer nuclear layer; INL, inner nuclear layer; IPL, inner plexiform layer; GCL, ganglion cell layer. Scale bar: 50 microns (A, C, E, G).

Adult retinas were characterized further using morphological analysis and antibody markers to identify cell types that express the *nrd:egfp* transgene. EGFP was observed in all rod photoreceptor cell bodies and in rod inner and outer segments (Fig. 7A and B′), but not in double cones (Fig. 7C and D′). Further, EGFP was observed in a subset of the amacrine cells, and very weak expression was detected in a small population of ganglion cells (Fig. 7E and F′), but not observed in Müller glia (Fig. 7G and H′). Since adult zebrafish contain at least 17 subtypes of bipolar cells, EGFP-positive bipolar cells were identified by the location, size and shape of the somata, shape of the dendritric tree, and the sublaminal innervation level in the inner plexiform layer (IPL). Based on the previously described characteristics of each subtype, we observed seven subtypes of EGFP-positive OFF bipolar cells (B_off_-s1, B_off_-s2w, B_off_-s3, B_off_-s1/s2, B_off_-s1/s3, B_off_-s2/s3, and B_off_-s1/s4) in adult *nrd:egfp* retinas, including many cases where the projections could be traced from the photoreceptors to the IPL (Fig. 7F′).

### Tg(*nrd:egfp*) expression in the light-damaged adult zebrafish retina

**Figure 8:**
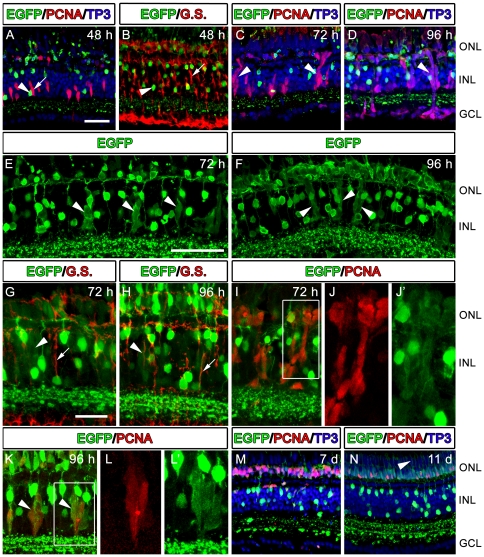
Retinal sections from adult Tg(*nrd:egfp*)/*albino* zebrafish over a time course of light treatment and immunolabeled with EGFP (green) to visualize the *nrd:egfp* transgene and co-labeled with either PCNA (A, C, D, I, J, J′, K, L, L′, M, N) or Glutamine Synthetase (B, G, H). (A) At 48 hours after light onset, almost all rod photoreceptors have been ablated and proliferating cells can be seen in the in the INL. Nuclei are labeled in blue with TO-PRO-3 (TP3). (B) At this time point, Müller glial cells express Glutamine Synthetase (G.S., red, arrow), and do not co-label with EGFP (arrowhead). (C) At 72 hours after light onset, clusters of proliferating progenitors begin to migrate towards the ONL (arrowheads). (D) At 96 hours post light onset, PCNA-positive progenitors (arrowheads) are present in both in INL and ONL, with occasional aberrant migration to the GCL. (E–F) At 72 and 96 hours after light onset, respectively, clusters of progenitors weakly express EGFP (arrowheads). (F) At 96 hours after light onset, EGFP is observed in a newly-formed and disorganized ONL. (G–H) At 72 and 96 hours after light onset, respectively, weakly-EGFP-positive clusters in the INL (arrowheads) down-regulated Glutamine Synthetase. Müller glial cells that did not re-enter the cell cycle strongly express G.S. (arrows), but are EGFP-negative. (I–J′) At 72 hours after light onset, weakly-EGFP-positive cells in both the INL and ONL co-label with PCNA. The box in I represents the PCNA and EGFP labeling shown in J and J′, respectively. (K–L′) At 96 hours after light onset, weakly-EGFP-positive cells in both the INL (arrowheads) and ONL continue to co-label with PCNA. The box in K represents the PCNA and EGFP labeling shown in L and L′, respectively. (M) At 7 days after light onset, a subset of PCNA-positive progenitors in the ONL co-label with EGFP (N) At 11 days after light onset, only a few PCNA-positive progenitors remain in the ONL. EGFP can be visualized in rod photoreceptors and newly-formed rod inner segments (arrowhead).

We examined the spatial and temporal expression of the *nrd:egfp* transgene following photolytic lesions and during photoreceptor regeneration. Specifically, we examined expression of the *nrd:egfp* transgene in relationship to retinal progenitors immunolabled with PCNA and Müller glia immunolabeled with Glutamine Synthetase. In the INL, 48 hours after light onset, Müller glial reenter the cell cycle and express PCNA (Fig. 8A; see Vihtelic and Hyde 2000). At this time, EGFP was not detected in the Glutamine Synthetase-positive Müller glial or their immediate progeny (Fig. 8A and B). At 72 and 96 hours after light onset, large numbers of progenitor cells were observed (Fig. 8C and D). Very weak EGFP expression was also observed in clusters of cells in the INL (Fig. 8E and F). Further characterization of these EGFP-positive clusters revealed a down-regulation of Glutamine Synthetase (Fig. 8G and H) and PCNA co-immunolocalization (Fig. 8I–L′). This is consistent with a previous report that showed that Müller glia down-regulate cell-specific markers after the re-enter the cell cycle to produce large clusters of PCNA-positive progenitors [32]. At 96 hours after light onset, weak, somewhat disorganized, EGFP-positive cells were present in the ONL (Fig. 8F), and co-labeled with PCNA (Fig. 8K). 7 days after light onset, proliferating cells in the INL were not observed, however, EGFP co-labeled with a large number of PCNA-positive progenitors in the ONL (Fig. 8G). 11 days after light onset, the transgene was weakly expressed in the newly formed rod photoreceptors in the ONL (Fig. 8H).

**Figure 9:**
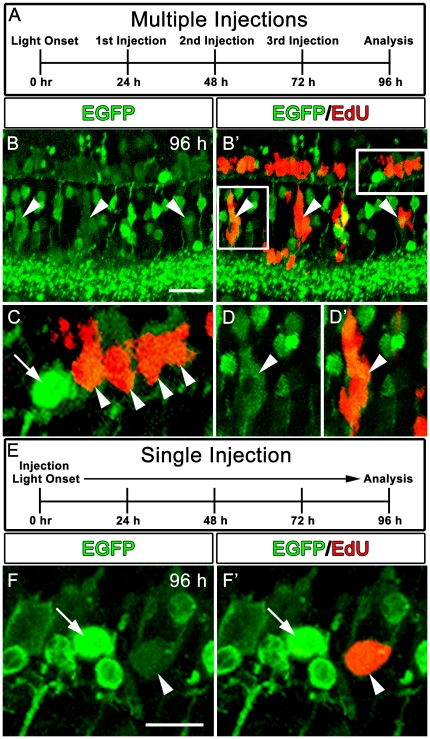
High magnification images of retinal sections from adult Tg(*nrd:egfp*)*/albino* zebrafish over a time course of light treatment. Sections were immunolabeled with EGFP (green) to visualize the *nrd:egfp* transgene and Zpr-3 (red) to visualize rod photoreceptors. (A) Prior to light treatment (0 hr), EGFP co-labels with Zpr-3 and is observed in rod photoreceptor soma, rod inner segments (RIS) and rod outer segments (ROS). (B) At 48 hours after light onset, the ROS and RIS are almost completely destroyed and only a few EGFP-positive cells remain in the ONL. (C) At 72 hours after light onset, newly-formed rod progenitor cells are present in the ONL. These could be readily discerned from existing rod photoreceptors due to their comparably weak expression of the transgene (inset shows new rod progenitor on the left). (D) At 96 hours after light onset, a greater number of new regenerated cells are present in the ONL, although it still somewhat disorganized. (E) At 7 days after light onset, newly differentiated rod photoreceptors appear more organized and greater in abundance. (F) At 11 days after light onset, EGFP is expressed in the newly formed rod photoreceptors and co-labels with Zpr-3-positive and newly-formed RIS and R0S. Scale bar: 50 microns (A–F).

A closer examination of the outer retina was performed using Zpr-3, which labels rod photoreceptor outer segments. 48 hours after light onset, the number of EGFP-positive rod photoreceptors was greatly reduced, along with their Zpr-3-positive outer segments (cf. Figs. 9A and 9B). By 72 hours after light onset, newly-formed rod progenitors were observed in the ONL (Fig. 9C). These could be readily discerned from existing rod photoreceptors due to their comparatively weak expression of the transgene (Fig. 9C, inset). 96 hours after light onset, the number of EGFP-positive rod progenitors was greatly increased, although they were still somewhat disorganized (Fig. 9D). 7 days after light onset, the newly formed rod photoreceptors had become more organized (Fig. 9E) and 11 days after light onset regenerated rod inner segments and Zpr-3-positive outer segments were observed (Fig. 9F). Full regeneration of rod outer segments was not achieved until 28 days after light onset (data not shown).

**Figure 10:**
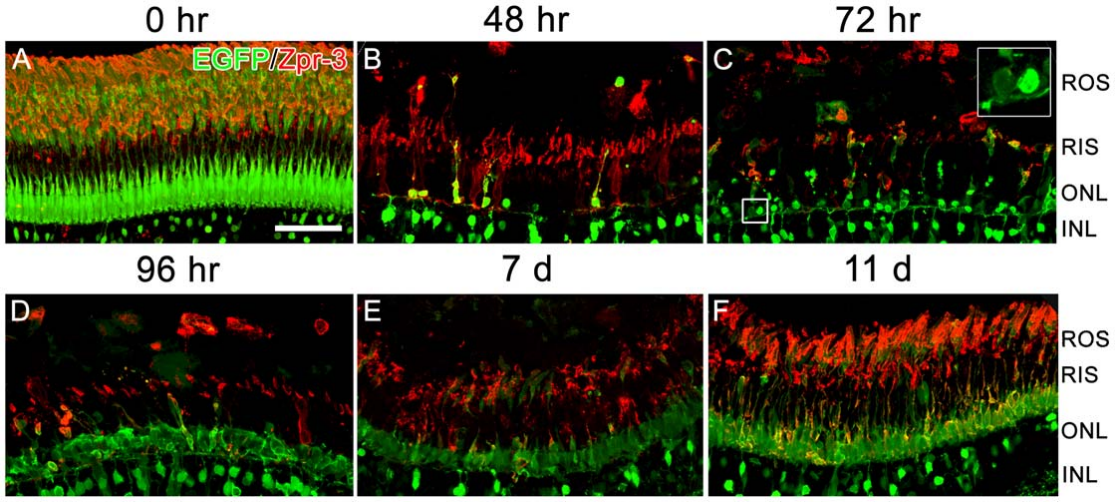
Retinal sections from adult Tg(*nrd:egfp*)/*albino* zebrafish at 96 hours after light onset showing transgene expression (green) and EdU labeling (red). (A). Schematic representation of EdU injections during the light time course with corresponding immunolocalization shown in Panels B–D′. (B–B′) EGFP and EGFP/EdU co-labeling, respectively, showing weakly-EGFP-positive cells in the INL (arrowheads) and ONL co-label with EdU. The boxes in B′ represent the panels shown in C–D′. (C) Higher magnification image of the box shown in the top right of Panel B′. Note that the weakly-EGFP-positive progenitors co-label with EdU (arrowheads), but strongly-EGFP-positive rod nuclei (arrow) are EdU-negative. (D–D′) Higher magnification image of the box shown in the left of Panel B′, showing EGFP and EdU immunolocalization, respectively, in a cluster of INL progenitors. (E) Schematic representation of a single EdU injection prior starting the light treatment in order to label a subset of the progenitors. (F–F′). High magnification confocal microscopy showing EGFP and EGFP/EdU co-labeling in the ONL at 96 hours after light onset. An individual EdU-positive cell in the ONL (arrowhead) co-labels with weak EGFP expression. The strongly-EGFP-positive cell, in contrast, is EdU-negative (arrow).

In order to determine whether the weakly-EGFP positive cells in the ONL (Fig. 9C, inset) were derived from progenitors or were undamaged photoreceptors that simply down-regulated EGFP, we performed an EdU labeling experiment. As was previously reported [35], daily injections of EdU following light onset results in labeling of many, if not all, of the neuronal progenitors. We repeated this method (Fig. 10A) and found that at 96 hours after light onset all the weakly-EGFP-positive cells in both the INL and ONL were also EdU-positive (Fig. 10B, B′). For a better resolution of individual cells in the ONL, we performed a single injection of EdU immediately prior to the light treatment, which only labeled a subset of the progenitors. At 96 hours after light onset, we found that the EdU-positive cells in the ONL were weakly stained with EGFP (Fig. 10F, F′), indicating that they were derived from progenitors. Importantly, with either injection method, we found that none of the strongly-EGFP-positive rod nuclei in the ONL were EdU positive (Fig. 10B, B′, F, F′), indicating that this line can be used to distinguish between undamaged and newly-formed rod photoreceptors.

### Tg(*nrd:egfp*) expression in comparison to endogenous *neurod* expression during photoreceptor regeneration

**Figure 11:**
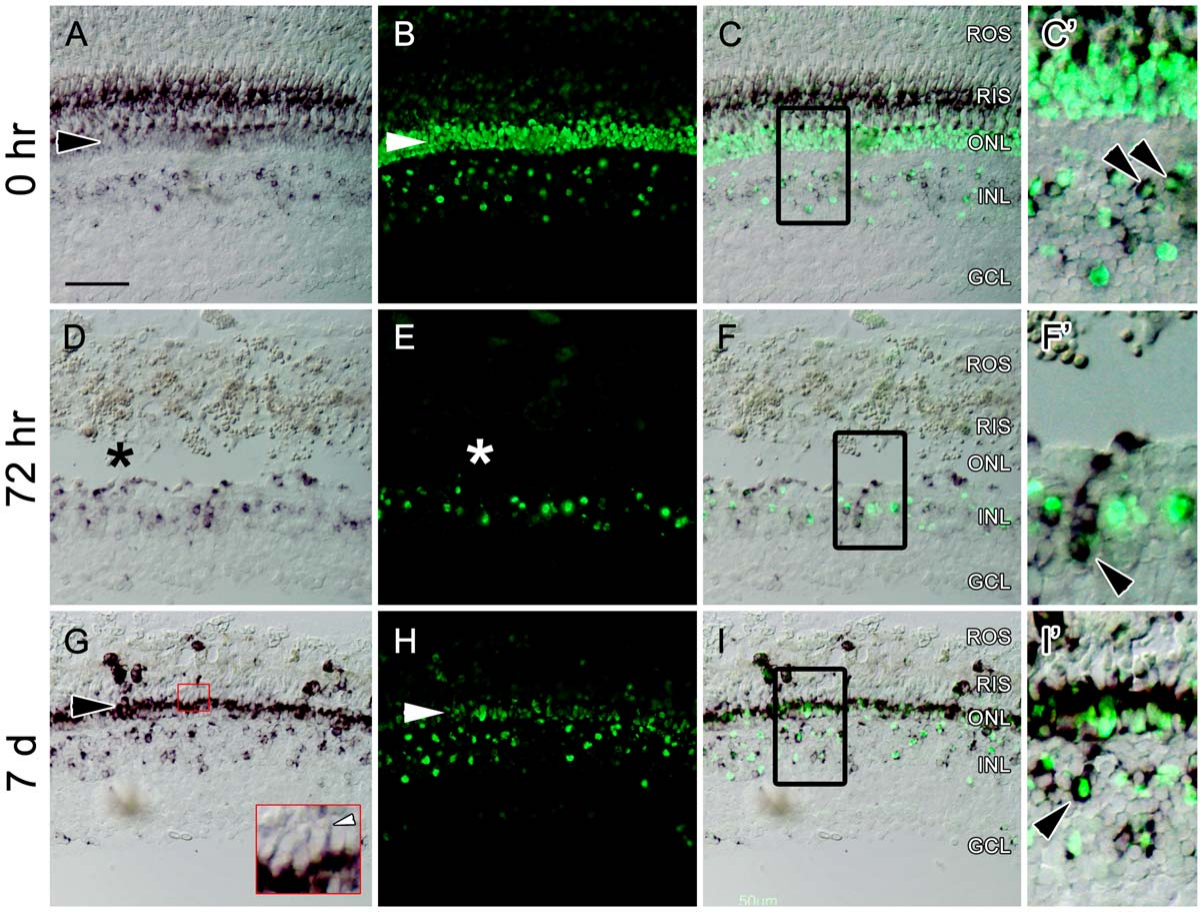
RNA *in situ* hybridization on retinal sections from adult Tg(*nrd:egfp*)/*albino* zebrafish comparing endogenous *neurod* expression (purple) to transgene expression (green) during light-induced retinal regeneration. (A) Before light treatment (0 hr), endogenous *neurod* is expressed in a subset of amacrine and bipolar cells, weakly expressed in rod photoreceptors in the ONL (arrowhead), and expressed in rod inner segments (RIS). (B) The *nrd:egfp* transgene is expressed in a subset of amacrine and bipolar cells, in rod photoreceptors in the ONL (arrowhead), and weakly in RIS. (C) Overlay of panels (A) and (B). (C′) Higher magnification inset of (C) showing co-labeling of endogenous and transgenic *neurod* expression in a subset of cells in the INL (arrowheads). (D) 72 hours after light onset (72 hr), all rod photoreceptors have been ablated (indicated by the asterisk), and endogenous *neurod* is persistently expressed in a subset of amacrine and bipolar cells. (E) The *nrd:egfp* transgene is persistently expressed in a subset of amacrine and bipolar cells. (F) Overlay of panels (D) and (E). (F′) Higher magnification inset of (F) showing co-labeling of endogenous and transgenic *neurod* expression in a column of progenitor cells (indicated by the arrowhead), and a subset of cells of the INL. (G) 7 days after light onset (7 d), endogenous *neurod* is strongly expressed in newly formed rods in the ONL (black arrowhead), and persistently expressed in a subset of amacrine and bipolar cells. The inset shows expression of *neurod* in newly-formed rod inner segments (white arrowhead). (H) The transgene is more weakly expressed in newly formed rods, and persistently expressed in a subset of amacrine and bipolar cells. (I) Overlay of panels (G) and (H). (I′) Higher magnification inset of (I) showing co-labeling of endogenous and transgenic *neurod* expression in a subset of INL cells (arrowhead), and in newly formed rod progenitors. Scale bar: 50 microns (A–C, D–F, G–I).

*In situ* hybridization was used to compare endogenous and transgenic expression of *neurod* during photoreceptor regeneration. Prior to light treatment, dark-adapted adult Tg(*nrd:egfp*)*/albino* retinas showed endogenous *neurod* in a subset of amacrine and bipolar cells in the INL, weak expression in rod photoreceptor soma, and strong expression in rod inner segments (Figs. 6I, 11A). The expression of endogenous *neurod* in the rod inner segments was not observed in non-dark treated animals (data not shown), indicating dynamic expression changes of *neurod* in photoreceptors during dark adaptation. Similarly, EGFP was strongly expressed in all rod photoreceptors, and a subset of amacrine and bipolar cells (Fig. 11B). 72 hours after light onset, nearly all rod and cone photoreceptors are destroyed (Fig. 10D and E, asterisk). Endogenous *neurod* was observed in isolated INL progenitors as they migrated to the ONL (Fig. 11F′). Weak EGFP expression was observed in these cells using GFP immunohistochemistry alone (Fig. 8E and F), but not when GFP immunohistochemistry was combined with *in situ* hybridizations. At 7 days after light onset, two distinct bands of endogenous and transgenic *neurod* were observed in the ONL (Fig. 11G and H). EGFP was observed in a band of the cell bodies of newly regenerated rods immediately adjacent to the outer plexiform layer (i.e. toward the inner retina) (Fig. 11I′). Endogenous *neurod* was strongly expressed in a band of rod cell bodies immediately distal to the EGFP band (Fig. 11I′), with only an occasional co-labeling among the cells residing in these two bands (Fig. 11I′).

## Discussion

To evaluate the utility of the *nrd:egfp* transgenic line, we compared the expression of the transgene to that of endogenous *neurod* during retinal development, in the adult retina and during photoreceptor regeneration. Previously, RNA *in situ* hybridization showed that during early retinogenesis *neurod* is first expressed in the ventral nasal patch and then throughout the neuroepithelium. Subsequently, *neurod* is transiently expressed in the nascent photoreceptors in the outer nuclear layer and persistently expressed in a subset of amacrine cells in the inner nuclear layer [1]. Similarly, we show that the *nrd:egfp* transgene is initially expressed adjacent to the ventral nasal patch (Figs. 1F and 6B), and then throughout the neuroepithelium and nascent photoreceptor layer (Fig. 6C). In contrast to the *in situ* data, however, EGFP is also present in bipolar cells, in a small fraction of rod photoreceptors at 2 wkpf, and in all rod photoreceptor cell bodies at adulthood.

There are potential explanations for the subtle temporal and cellular disparities in the expression of *neurod*, as detected by *in situ* hybridizations, and the expression of the *nrd:egfp* transgene. One possibility is that the *neurod* transgene lacks a required silencer or is influenced by neighboring enhancers near the site of integration. However, it would have to lie far outside the coding region, as the transgene contains 67 kb of sequence upstream and 89 kb of sequence downstream of *neurod* open reading frame [33]. Another possibility is that mature bipolar cells and rod photoreceptors, not observed following *in situ* hybridizations, produce very low levels of endogenous *neurod*, and the stability of EGFP more readily allows for the detection of these cells. In support of this interpretation, prior to light treatment we observed weak expression of endogenous *neurod* in all rod photoreceptor cells by *in situ* hybridization, and strong expression of EGFP in the same cells (Figs. 6I–I″′, 11A and B).

We observed both overlapping and distinct expression profiles for endogenous and transgenic *neurod* expression during retinal regeneration. In both cases, *neurod* was not observed in dividing Müller glia or in the early stages of neuronal progenitor amplification. Both endogenous and transgenic *neurod* were first observed in INL progenitors in later stages of proliferation as these progenitors were migrating to the ONL (Fig. 11F′ and 8E). At this point endogenous *neurod* expression is very strong in these progenitors, whereas EGFP is very weak (cf. Figs. 11F′and 8E). By 3 days post light treatment, two distinct bands of expression were observed. At this point, endogenous *neurod* is downregulated in the first wave of newly regenerated rod photoreceptors that are closest to the INL, whereas EGFP was strongly expressed in these cells. In contrast, endogenous *neurod* is highly expressed in the next wave of rod photoreceptors located distal to the first band of cells, but EGFP is not yet present. These differences in endogenous and transgene expression may be explained by dynamic changes in endogenous *neurod* expression compared to the relatively long (∼24 hour) half-life of EGFP. In each case, endogenous *neurod* expression proceeded EGFP expression and EGFP was visualized after the downregulation of endogenous *neurod*.

Expression of *neurod* is often found in tissues with persistent mitotic activity. Although the zebrafish retina continues to grow throughout its life, we did not observe the *neurod* transgene in known locations of persistent neurogenesis in the retina. For example, consistent with previously published *in situ* hybridizations, *neurod* transgene expression was not observed during retinogenesis in the progenitors located in the circumferential marginal zone (CMZ), but did overlap with PCNA-positive cells as they exit the CMZ and begin differentiating (Fig. 5A). Similarly, during retinal regeneration, endogenous and transgenic *neurod* was not observed in Müller glial or their immediate progeny, but in later stage progenitors prior to photoreceptor differentiation (Figs. 8, 10, 11). This is consistent with anti-sense morpholino studies in early zebrafish development which show that in the absence of NeuroD, rod and cone progenitors fail to exit the cell cycle [20]. In addition, the developing chick retina requires *neurod* for photoreceptor differentiation [18], [19]. Together, these data suggest that the major function of NeuroD in the developing retina is in regulating mechanisms that promote cell cycle exit. It has yet to be determined whether NeuroD plays a similar role during retinal regeneration in the adult.

One potential use would be to utilize the line to visualize the reestablishment of the synapses connecting rod photoreceptor and bipolar cells. During intense light damage, rod photoreceptors are lost, but the underlying bipolar cells remain (Fig. 9B). Once disconnected from the photoreceptor, the bipolar cell processes hypertrophy and bud out, presumably in an attempt to re-establish the lost connection (data not shown). Once the new photoreceptor is regenerated, this connection is re-established. Since a subset of bipolar cells and newly formed rod photoreceptors are both EGFP-positive, this line could be used for *in vivo* imaging and genetic manipulation of this dynamic and poorly understood process.

This line also has potential uses for studies on the endocrine pancreas. NeuroD has been shown to be expressed in the endocrine pancreas in a variety of vertebrates [5], [41]. Loss of NeuroD in mice results in abnormal pancreatic β-cell maturation and function [42], severe hyperglycemia and neonatal death [43]. We show the *neurod* transgene is expressed the endocrine pancreas and could be used as a visual marker for β-cell function, particularly in the growing field using zebrafish as a vertebrate model for diabetes [44], [45], [46].

In summary, given the diverse areas of *neurod* expression in the developing and adult zebrafish, we anticipate that the Tg(*nrd:egfp*)*/alb* line will be a useful tool in multiple disciplines, including future studies on photoreceptor differentiation and retinal progenitor proliferation.
